# Dentine biomodification by sulphonamides pre-treatment: bond strength, proteolytic inhibition, and antimicrobial activity

**DOI:** 10.1080/14756366.2022.2150184

**Published:** 2022-11-28

**Authors:** Maristela Barbosa Portela, Caroliny Mello Barboza, Eduardo Moreira da Silva, Daniel Clemente de Moraes, Renata Antoun Simão, Clara Ribeiro de Souza, Verônica da Silva Cardoso, Antônio Ferreira-Pereira, Alane Beatriz Vermelho, Claudiu T. Supuran

**Affiliations:** a Departamento de Odontotécnica, Laboratório Analítico de Biomateriais Restauradores (LABiom-R), Faculdade de Odontologia, Universidade Federal Fluminense, Niterói, Brazil; b Laboratório de Bioquímica Microbiana, Instituto de Microbiologia Paulo de Góes, Universidade Federal do Rio de Janeiro, Rio de Janeiro, Brazil; c Instituto Alberto Luiz Coimbra de Pós-Graduação e Pesquisa de Engenharia (COPPE), Universidade Federal do Rio de Janeiro, Rio de Janeiro, Brazil; d Bioinovar-Biotecnologia: Unidade de Biocatálise, Bioprodutos e Bioenergia (BIOINOVAR), Instituto de Microbiologia Paulo de Góes, Universidade Federal do Rio de Janeiro, Rio de Janeiro, Brazil; e NEUROFARBA Department, Sezione di Scienze Farmaceutiche, Universita degli Studi di Firenze, Florence, Italy

**Keywords:** Carbonic anhydrase inhibitor, dentine, metalloproteinases, antimicrobial activity, *Streptococcus mutans* biofilm

## Abstract

We evaluated the effects of dentine biomodification after pre-treatment with two sulphonamide carbonic anhydrase inhibitors (CAIs) of the N-[4-sulphamoylphenethylcarbamoyl]benzenesulphonamide type, investigating matrix metalloproteases activity, resin–dentine micro tensile bond strength, dentine surface wettability, and antimicrobial activities. Ninety-five sound-extracted human molars were selected for the study. Inhibitory effects were evaluated by gelatinase and collagenase activity tests and collagen degradation FT-IR spectroscopic analysis. Pre-treatment with the two CAIs kept the micro tensile values after 12 months of storage (32.23 ± 5.95) and cariogenic challenge (34.13 ± 2.71) similar to the initial, pre-treatment values (33.56 ± 4.34). A decreased *Streptococcus mutans* biofilm formation on dentine surfaces and antibacterial activity against planktonic bacteria were observed after CAI treatment. Dentine pre-treatment with sulphonamide CAIs maintained adhesion strength stability, allowed better dentine wettability, maintained matrix collagen, and showed anti-*S. mutans* activity.

## Introduction

Despite the constant and visible advances regarding the physicochemical characteristics of resin composites and their adhesion to dental tissues, it is still possible to observe failures in the resin–dentine bonding interface over time^1^^,^^2^. For the Effectiveness of adhesive procedures, dentine’s structure, composition, and characteristics are decisive since adhesion to the dentine substrate is more complex and challenging than to the enamel^3^. In general, dentine is a mineralised tissue based on hydroxyapatite and collagen embedded in an extracellular matrix. Approximately 10% of this matrix comprises proteins, including proteoglycans and enzymes^4^.

Among these enzymes, the matrix metalloproteinases (MMPs) are involved in collagen degradation in the hybrid layer and the progression of dentinal caries^5^^,^^6^. The collagen degradation occurs due to the incomplete impregnation of resin monomers in demineralised dentine by phosphoric acid conditioning or acidic monomers present in adhesive systems, creating discrepancies between demineralisation depth and monomeric infiltration, leading the collagen fibres unprotected and activating the MMPs^2–4^. In the progression of dental caries, the acidic environment created by the cariogenic biofilm generates the dissolution of the inorganic content of dental tissues exposing the organic portion constituted by collagen fibres. The action of the MMPs degrades these unprotected fibres activated due to the low pH^7–9^.

Studies have been developed to identify substances capable of inhibiting the activity of MMPs. Inhibitors such as chlorhexidine and galardin^3^^,^^4^^,^^10–14^ can improve the integrity and stability of the dentine–resin bond when used as a dentine pre-treatment. However, the results of the usage of these inhibitors in dentine quality are still controversial. Among these inhibitors, chlorhexidine is the most frequently investigated one, being able to reduce the degradation at the dentine–resin interface^11^^,^^15^. Nevertheless, this inhibitor is susceptible to long-term leaching, interrupting the inactivation of the MMPs, promoting the degradation of collagen fibrils exposed at the adhesive interface, and decreasing the resin–dentine bond strength^16^.

In addition to the presence of MMPs in dentine, these enzymes are also found in other parts of the human body and other organisms, being involved in a broad spectrum of important biological reactions, including protein metabolism, immune reactions, tissue remodelling, and blood clotting^17^. Thus, the cellular proteolytic activity must be highly regulated to prevent inappropriate and uncontrolled degradation of proteins. As a result, several studies have been conducted to discover alternative inhibitors of these MMPs^18–23^.

Sulphonamides and their derivatives are efficient inhibitors of carbonic anhydrases (CAs), metalloenzymes that catalyse the reaction between CO_2_ and water, generating H^+^ and HCO_3_^−^^24–28^. Nonetheless, to our knowledge, no reports show these compounds act as inhibitors of enzymes/processes that improve dentine adhesion. Thus, this present study aimed to evaluate the protective effects of sulphonamide CA inhibitors (CAIs) on dentine. The tested hypotheses were: (1) the pre-treatment of the dentine surface with sulphonamide derivatives will not decrease the micro tensile strength after ageing in distilled water for 12 months and after cariogenic challenge, and it will not influence dentine wettability as well; (2) the sulphonamide derivatives will be capable of inhibiting the proteolytic activity of the dentine matrix enzymes with the preservation of dentine collagen; and (3) the sulphonamide derivatives will present antimicrobial and antibiofilm activity against *Streptococcus mutans*, one of the bacterial species present in the oral cavity, probably due to inhibition of the β-class CA present in this pathogen^25^.

## Materials and methods

### Study design and materials

The present study was approved by the Ethics and Research Committee of the Hospital Universitário Antônio Pedro (Universidade Federal Fluminense, RJ, Brasil) under the number 3.383.557.

Table 1 describes the composition and manufacturers of all commercial materials used in the present study.

**Table 1.** Composition and manufacture of commercial materials used at the study.

Material / commercial name	Composition	Manufacturer
Phosphoric acid / Ultra-Etch	Phosphoric acid 37%	(Ultradent Products Inc.– SouthJordan, USA)
Adhesive system / Adper Single Bond 2	BisGMA (10–20%), HEMA (5–15%), ethanol (25–30%), water (<5%), glycerol 1,3-dimethacrylate (5–10%), urethane dimethacrylate (UDMA) (5–15%), Copolymer of acrylic acid and itaconic acid, silica-silicate (10–20% nanoparticulate), diphenyliodonium hexafluorophosphate	(3M ESPE, StPaul, MN, USA)
Composite / Filtek Z250	Zirconia/Silica, BIS-GMA, BIS-EMA, TEGDMA and UDMA.	(3M ESPE, St Paul, MN, USA)

### Chemicals

**Figure 1.**
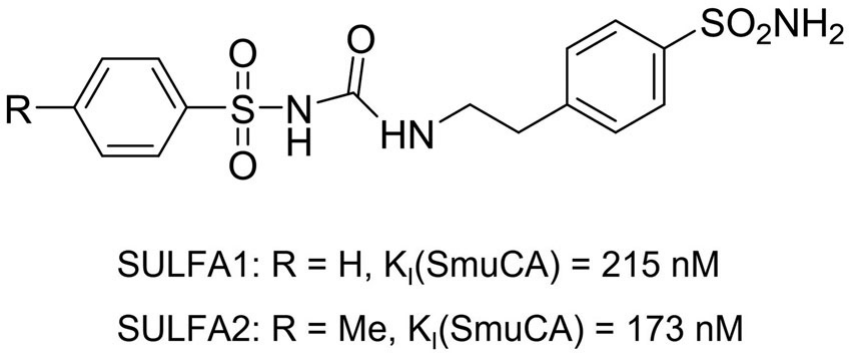
Chemical structures of the two arylsulphonyl-ureido-benzenesulphonamides SULFA1 and SULFA2 and their inhibitory activity against *S. mutans* β-CA (SmuCA).

The synthesis of sulphonamide derivatives was previously described^29^^,^^30^ and consisted in reacting aminosulphonamides with aryl sulphonyl isocyanates, originating in a series of aryl sulphonyl-ureido aromatic/heterocyclic sulphonamides, two of which, namely, N-[4-sulphamoylphenethylcarbamoyl] benzenesulphonamide (SULFA1) and 4-methyl-N-[4-sulphamoylphenethylcarbamoyl] benzenesulphonamide (SULFA2) have been employed in the work (see Figure 1 for structural details). SULFA1 and SULFA2 were solubilised in dimethyl sulphoxide (DMSO) (Sigma-Aldrich®, St. Louis, MO, USA) and then diluted in distilled water until reaching concentrations of 100, 150, and 200 µM. 1,10-phenanthroline (Sigma-Aldrich®, St. Louis, MO, USA) at 100 µM was used as a standard metalloprotease inhibitor.

### Gelatinolytic and collagenolytic activity

The concentration of SULFA1 and SULFA2 derivatives used in the present study was previously defined through an experiment that aimed to verify the concentration with the best inhibitory proteolytic activity of the MMPs^31^.

The gelatinolytic and collagenolytic activity of SULFA1 and SULFA2 were assessed as described by Li et al^32^. Human molars teeth (n-5) without dental caries extracted due to dental need were cleaned with periodontal curettes, stored for seven days in 0.5% chloramine aqueous solution for disinfection, and kept in distilled water under refrigeration before use. The occlusal enamel was removed in a metallographic cutter (ISOMET 1000, Buehler Ltd, IL, USA). The marginal enamel was removed with a diamond tip (4138, KG Sorensen, Curitiba, PR, BR) assembled on a high-speed turbine under refrigeration. Following this step, teeth were sectioned at the level of the cement-enamel junction, and enamel and pulp tissues were removed. The dentine blocks were frozen in liquid nitrogen to be crushed in a ball mill (Model MM400, Retsch GmbH, Haan, DE) at 30 Hz/7 min. The dentine powders were then demineralised with 37% phosphoric acid for 15 s and rinsed with distilled water. After drying, three different concentrations of the compounds were added: 100 μM, 150 μM, and 200 μM. Eighty milligrams of dentine powders and 20 µl of 100 µg/mL gelatin/collagen (EnzChek-E-12055, Molecular Probes, Eugene, OR, USA) were added to 96-well plates and incubated at 37 °C for 1 h. Fluorescent cleavage products, derived from the proteolytic activity, were measured using a microplate reader (FLUOstar Optima®, BMG Labtech, Ortenberg, GER) with maximum absorption of 495 nm and maximum fluorescence emission of 515 nm. Each well’s relative fluorescence unit (RFU) was corrected by subtracting the background fluorescence value of the non-enzyme control; 2% DMSO (Sigma-Aldrich®, St. Louis, MO, USA) was used as control.

### Assessment of dentine adhesion

#### Specimens preparation

This assay used 50 donated human molars with no cavities and extracted due to dental need. The teeth were cleaned with periodontal curettes, stored for seven days in 0.5% chloramine aqueous solution for disinfection, and kept in distilled water under refrigeration until their use. The teeth were included by the roots with acrylic resin within the PVC rings. After polymerisation of the acrylic resin, the occlusal enamel was removed in a metallographic cutter (ISOMET 1000, Buehler Ltd, IL, USA), exposing the superficial layer of dentine. Then, the marginal enamel was removed with a diamond tip (4138, KG Sorensen, Curitiba, PR, BR) assembled on a high-speed turbine under refrigeration. The dentine smear layer was standardised with 150, 400, and 600-grit SIC papers in a polishing machine (DPU 10, Struers, DEN) for 1 min. After the preparation of the dentine surfaces, the teeth were randomly divided into the following experimental groups (*n* = 5): SULFA1 − 37% Phosphoric Acid + 200 µM N-(4-sulphamoyl phenethyl carbamoyl) benzenesulphonamide + Adper Single Bond 2; SULFA2 − 37% Phosphoric Acid + 200 µM 4-Methyl-N-(4-sulphamoyl phenethyl carbamoyl) + Adper Single Bond 2; DMSO − 37% Phosphoric Acid + 2% dimethyl sulphoxide + Adper Single Bond 2; PHEN − 37% Phosphoric Acid + 1,10-phenanthroline + Adper Single Bond 2; CTR 37% Phosphoric Acid + Adper Single Bond 2.

The etching of the dentine with 37% phosphoric acid occurred for 15 s, followed by washing with water for 30 s and drying with absorbent paper. Dentine hybridisation with Adper Single Bond 2 adhesive followed the manufacturer’s recommendations. Subsequently, Filtek Z250 composite blocks with 5.0 mm high were constructed incrementally on the dentine surface. Each increment was photoactivated with an irradiance of 1000 mW/cm^2^ for the 20 s (DEMI, Demetron Inc., Danbury, USA). After the composite block was made, the teeth were stored in distilled water at 37 °C for 24 h. Thus, the teeth were taken to the metallographic cutter (IsoMet 1000, Buëhler, Lake Bluff, IL, USA) and serially sectioned with a double-sided diamond disc under refrigeration in two planes perpendicular to the adhesive interface, obtaining beams with approximately 1.0 mm^2^ cross-section. After the cut, all beams were evaluated in a stereomicroscope (SZ40, Olympus, Tokyo, JPN), with a 40× magnification, to discard those with structural defects (cracks or blisters in the composite).

The beams obtained from each tooth for each group were randomly divided into three subgroups: (1) 24 h of immersion in distilled water (SULFA1 24H, SULFA2 24H, DMSO 24H, CTR 24H E PHEN 24H); (2) 12 months of immersion in distilled water (SULFA1 12 M, SULFA2 12 M, DMSO 12 M, CTR 12 M E PHEN 12 M) at 37 °C. The distilled water was replaced weekly for fresh amounts; (3) submitted to *S. mutans* biofilm for 48 h at the cariogenic challenge (CC) (SULFA1 CC, SULFA2 CC, DMSO CC, CTR CC E PHEN CC).

The beams submitted to the cariogenic biofilm were painted with nail varnish (Colorama-CIEIL-SP, BR), leaving 1.0 mm^2^ free around the bonded interfaces. The specimens were then fixed in 24-well plates (TPP, 24 Zellkultur Festplatte F, GER). The cariogenic challenge was performed according to Carvalho et al^33^. Afterwards, the beams were exposed to ultraviolet light for disinfection^34^ and subjected to bacterial suspension of *S. mutans* (ATCC 25175), prepared as follows: the *S. mutans* strain was cultured in Mitis Salivarius agar (Difco, Sparks, USA) at 37 °C under low oxygen tension conditions. Subsequently, single colonies were inoculated into 5 ml of Brain Heart Infusion (BHI) broth (Difco, Sparks, USA) supplemented with 0.5% glucose and 1.0% sucrose and incubated at 37 °C for 24 h. The bacterial suspension was adjusted to 2 × 10^8^ CFU/ml. During the experiment, the growth environment was changed every 24 h. The beams remained for three days at 37 °C under low oxygen conditions.

#### Microtensile bond strength (µTBS)

The cross-sectional area of the beam was measured with a digital calliper (MPI/E-101, Mitutoyo, Tokyo, JAP). Then, they were fixed to a micro tensile device (ODMT03d, Odeme Biotechnology, Santa Catarina, BRA) using cyanoacrylate glue (Super bonder Gel, 3 M, São Paulo, BRA) and loaded under tension in a universal testing machine (EMIC DL 2000, São José dos Pinhais, São Paulo, BRA) at a crosshead speed of 1.0 mm/min until failure occurred. The µTBS (MPa) was obtained by dividing the load at failure (N) by the cross-sectional area (mm^2^). An average was calculated with the bond strength values presented by the beams obtained for each tooth so that the tooth was considered the experimental unit for the statistical test.

#### Failure mode

The fractured surfaces were evaluated under a stereomicroscope at 40x magnification (SZ40, Olympus, Tokyo, Japan). The mode of failure was classified as an adhesive (failures at the adhesive interface), cohesive (failures occurring within dentine or resin composite), or mixed (mixture of adhesive and cohesive failure within the same fractured surface).

### Contact angle measurements

To evaluate the contact angle, 25 dentine discs 1.0 mm thick (from 17 human molars) were made following the methodology described by Ricci et al^35^. The specimens were etched with 37% phosphoric acid for 15 s, rinsed for 30 s with distilled water, and the blot was dried with absorbent paper. The specimens were randomly divided into groups according to dentine surface treatment (*n* = 5): SULFA1 – 200 µM, SULFA2 – 200 µM; DMSO – 2%; PHEN – 100 µM; CTR – without treatment. The contact angles of the specimens were measured using a contact angle goniometer (Model 200, Ramé-hart Instrument Company, Netcong, USA). On the dentine surface, 2.0 µl of the Adper Single Bond 2 adhesive system (3 M ESPE, St. Paul, MN, USA) was applied with a pre-adjusted 1.0 ml syringe on the dentine surface, followed by the measurement of the contact angle between the dentine surface and the liquid drop. Thirty measurements with an interval of 1 s were performed with the goniometer. Based on the data obtained for the liquid (DA), the Ramé-Hart software calculated the contact angle of the determined groups.

### Collagen degradation spectroscopic analysis

After wet grinding the coronal enamel (180-grit SiC paper) of three sound human molars until exposing flat dentine and removal of peripheral enamel, a 1.0 mm thick underlying dentine slice was obtained using a diamond saw in a cutting machine (ISOMET 1000, Lake Bluff, IL, USA) under water cooling. Then, the dentine slices were ground (600-grit Sic paper) until 0.5 mm thick dentine slices were obtained and monitored with a digital calliper (MPI/E −101, Mytutoyo, Tokyo, Japan). Each slice was immersed in 3 ml of 10% phosphoric acid and maintained for 40 h on a magnetic stirrer (Q261A11, Quimes, Rio de Janeiro, RJ, Brasil) until demineralisation and collagen loss. After washing for 10 min in distilled water, each demineralised dentine slice was cut with a scalpel blade to obtain collagen fragments (2 × 2 mm), which were assigned into five groups (SULFA1, SULFA2, DMSO, PHEN, and CTR). After immersion into groups for 1 min, the collagen fragments were gently dried with absorbent paper and stored in a collagenase solution from *Clostridium histolyticum* (Type I, 125 CDU/mg, Sigma-Aldrich®, St Louis, USA) (100 µg/ml) for 15 days at 37 °C, which was renewed daily.

The collagenase solution was prepared and renewed every 24 h from the solubilisation of 100 µg of collagenase from *C. histolyticum* (Type I, 125 CDU/mg, Sigma-Aldrich®, St Louis, USA) in 1 ml of buffer solution TESCA (5,72 g of acid N-Tris (hydroxymethyl) methyl-2-aminoethanesulphonic (Sigma-Aldrich®, St Louis, USA) and 20 mg of calcium chloride (Dinâmica, Indaiatuba, SP, BRA) dissolved in 500 ml of distilled water. All components were weighed on an analytical balance (XP 205, METTLER TOLEDO, Greinfensee, CHE) and then mixed manually.

The collagen was analysed according to Kishen et al.^36^ method, using an ATR crystal of an FT-IR (Alpha-P/Platinum ATR Module, Bruker Optics GmbH, Ettlingen, GER). The spectra were recorded between 1200 and 1800 cm^−1^ using 80 scans at a 4.0 cm^−1^ resolution after two storage periods: immediate (after application of the inhibitor for 1 h) and 15 days of storage. The infra-red bands considered for this study were 1648 cm^−1^ (amide I) and 1563 cm^−1^ (amide II).

### Antibacterial activity of CA inhibitors

For this assay, 20 sound molars were used to make 20 standardised dentine discs (5.0 × 5.0 mm and 1.0 mm thick). Discs were exposed to ultraviolet light for disinfection^34^, and the substances tested were previously sterilised by filtration. The discs were randomly divided into SULFA1, SULFA2, DMSO, PHEN, and CTR groups (*n* = 4). Then, 50 µl of the protease inhibitor solutions were applied to the dentine surface with a sterile micro applicator for 60 s, and excesses were removed with sterile absorbent paper. The pre-treated dentine discs were placed in 24-well plates, with the treatment surface facing up. After the growth of *S. mutans* in BHI for 48 h at 37 °C under low oxygen tension, the bacterial suspension was adjusted for an optical density of 0.5 (550 nm) according to the McFarland scale. Then, the suspension was diluted (1:100), 10.0 µl was diluted in 2 ml of BHI supplemented with 2% sucrose and added to each well with dentine discs. The 24-well culture plates were incubated at 37 °C under low oxygen tension for 48 h. Over the two days of biofilm formation, the culture environment was replaced every 24 h^37^.

The cell viability of the *S. mutans* biofilms on the adhesive discs was analysed by tetrazolium azide (MTT; Sigma-Aldrich®, St. Louis, MO, USA) reduction assay. Briefly, 1.0 ml of sterile MTT (1.0 mg/ml in PBS) was added to each well and incubated at 37 °C under low oxygen tension conditions for 1 h. Afterward, 1.0 ml of DMSO was added to each well, and the plates were incubated for 20 min at room temperature, protected from light, and with gentle agitation. Absorbance was obtained using a microplate reader at 540 nm.

In addition to the viability of the biofilm formed on the dentine blocks, the cell growth of *S. mutans* was measured. After 48 h of biofilm induction on the dentine blocks, 100 µl of the cell suspension from each well was transferred to 96-well plates, and absorbance at 660 nm was obtained to evaluate the turbidity of the suspension^38^.

### Statistical analysis

Statistical analysis was performed using Statgraphics Centurion 16.1 (Manugistics, Rockville, MD, USA). Initially, Shapiro-Wilk and Levene tests were applied to verify the normality of the distributions and the homoscedasticity of the variances. Based on the results, data regarding contact angle and antibacterial activity of protease inhibitors were analysed using the one-way analysis of variance (ANOVA). Data related to bond strength and gelatinolytic and collagenolytic activity were analysed using the two-way ANOVA. The Tukey HSD test was performed to contrast the means for both analyses. The failure mode and collagen degradation spectroscopic were evaluated qualitatively by the spectra analysis. All analyses were performed at a significance level of α = 0.05.

### *In vitro* CA inhibition assay

The inhibitory activity of the two sulphonamides against *S. mutans CA* (SmuCA) was assayed using Khalifah’s stopped flow assay^39^.

## Results

### Gelatinolytic and collagenolytic activity

By measuring the proteolytic activity through the fluorescence emitted by the cleaved EnzChek substrate, it was possible to determine that, for both substances (SULFA1 and SULFA2), the concentration of 200 µM was more effective in inhibiting collagen proteolysis since the results were statistically equal to the positive control group PHEN. Moreover, both compounds in all concentrations tested (100 µM, 150 µM, and 200 µM) presented a lower proteolytic activity when compared to the CTR group (Table 2).

**Table 2.** Relative fluorescence intensity emitted by the action of dentine gelatins.

Groups	Relative fluorescence intensity
*SULFA1 100*	*2,02±(0,26)^D^*
*SULFA1 150*	*1,78±(0,32)^CD^*
*SULFA1 200*	*1,21±(0,21)^AB^*
*SULFA2 100*	*1,58±(0,12)^BCD^*
*SULFA2 150*	*1,47±(0,14)^ABC^*
*SULFA2 200*	*1,06±(0,09)^A^*
*DMSO*	*7,38±(0,25)^E^*
*PHEN*	*0,98±(0,07)^A^*
*CTR*	*7,40±(0,59)^E^*

*Note:* In each column, groups followed by different capital letters are statistically different (HSD of Tukey, *p* < 0.05).

### Microtensile bond strength

The values of micro tensile bond strength (µTBS) of the experimental groups after different forms of ageing (24 h and 12 months in distilled water) and cariogenic challenge (CC) are shown in Table 3. The adhesion strength of the CTR group after CC (21.87 ± 3.74 MPa) and 12 months in distilled water (19.35 ± 2.19 MPa) were lower when compared to CTR after 24 h (31.91 ± 4.06 MPa). The SULFA1 and SULFA2 groups showed no decrease in bond strength values after 12 months of immersion in water or cariogenic challenge.

**Table 3.** Average values (± DP) of bond strength after storage in distilled water (24 h and 12 months) and cariogenic challenge (CC) by microtensile bond strength assay (MPa).

Groups	24 h in distilled water µTBS (MPa)	12 months in distilled water µTBS (MPa)	Cariogenic challenge (CC) µTBS (MPa)
SULFA1	33,56±(4,34)^Aa^	32,23±(5,95)^Aa^	34,13±(2,71)^Aa^
SULFA2	28,42±(3,50)^ABab^	24,09±(5,26)^ABa^	33,44±(1,65)^Ab^
DMSO	23,40±(3,56)^Ba^	21,60±(4,60)^Ba^	27,00±(2,55)^Ba^
PHEN	30,51±(5,45)^ABa^	20,78±(2,58)^Bb^	34,44±(3,55)^Aa^
CTR	31,91±(4,06)^Ab^	19,35±(2,19)^Ba^	21,87±(3,47)^Ba^

*Note:* Different capital letters vertically (column) indicate statistical difference between groups in the same column. Different lowercase letters horizontally (line) indicate statistical difference in the same group in different environments and storage times (24 h, 12 months, and cariogenic challenge).

It may be observed that after 24 h in distilled water and the cariogenic challenge, results obtained with SULFA1 and SULFA2 were comparable to the PHEN group (pre-treatment with phenanthroline, a standard inhibitor of metalloproteases). Interestingly, only SULFA2 was comparable to the PHEN group after one year of ageing in water. The DMSO group after CC had a lower bond strength value when compared to SULFA1 and SULFA2. None of the groups had premature failures during beams preparation or the µTBS test.

### Failure mode

**Figure 2.**
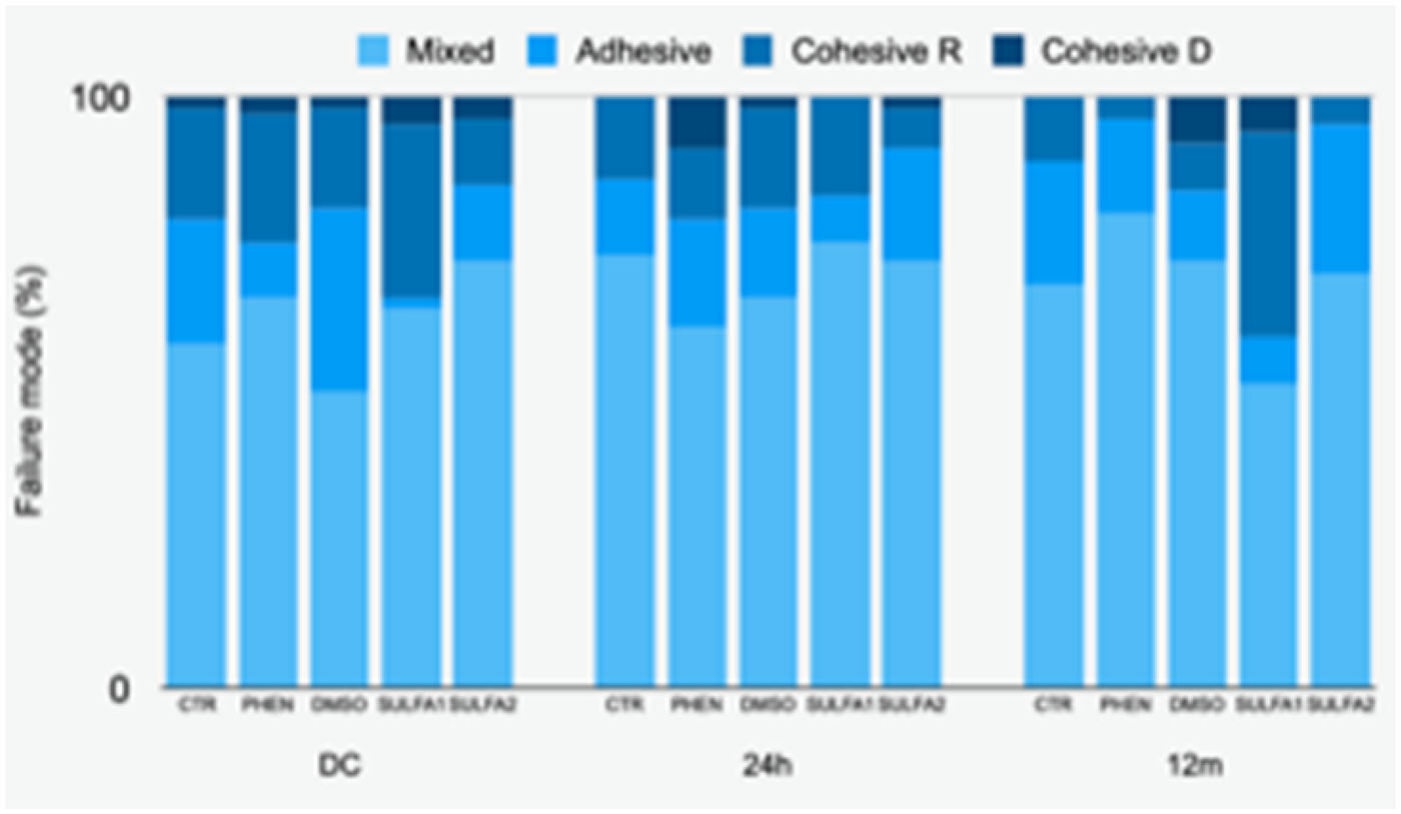
Graphical representation of failure patterns (%) after storage in distilled water for 24 h, 12 months and after cariogenic challenge (CC).

Regarding the analysis of the failure pattern after micro tensile in 24 h, there was a significant predominance of mixed failure in all groups and a significant number of adhesive failures in the PHEN group. After 12 months, although the predominance of failures in the groups continued to be mixed, there was a greater number of cohesive failures in resin in the SULFA1 group. After the cariogenic challenge, there was a predominance of mixed failures for all groups and a greater number of adhesive and cohesive failure in dentine for the PHEN group (Figure 2).

### Contact angle measurements

**Figure 3.**
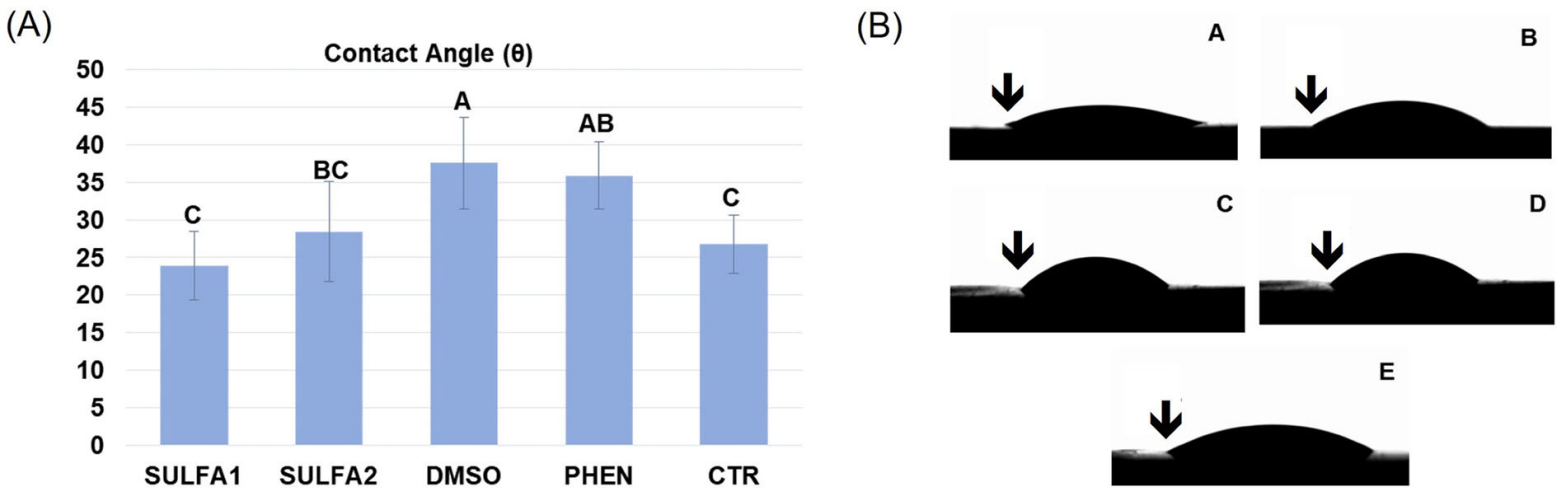
(A) Average values (±DP) of the contact angle. Slashes by different capital letters are statistically different (HSD of Tukey, *p* < 0.05). (B) Representative images of the groups: A-SULFA1; B-SULFA2; C-DMSO; D-PHEN; E-CTR. ↓ drop of adhesive in contact with the dentine, forming the contact angle.

The contact angle values and representative images of each group are depicted in Figure 3. This analysis showed that the wettability of the dentine surface pre-treated with the substances SULFA1 and SULFA2 was statistically equal to the CTR group. The SULFA1 group presented a smaller contact angle when compared to the PHEN group (*p* < 0.05).

### Spectroscopic analysis of collagen degradation

**Figure 4.**
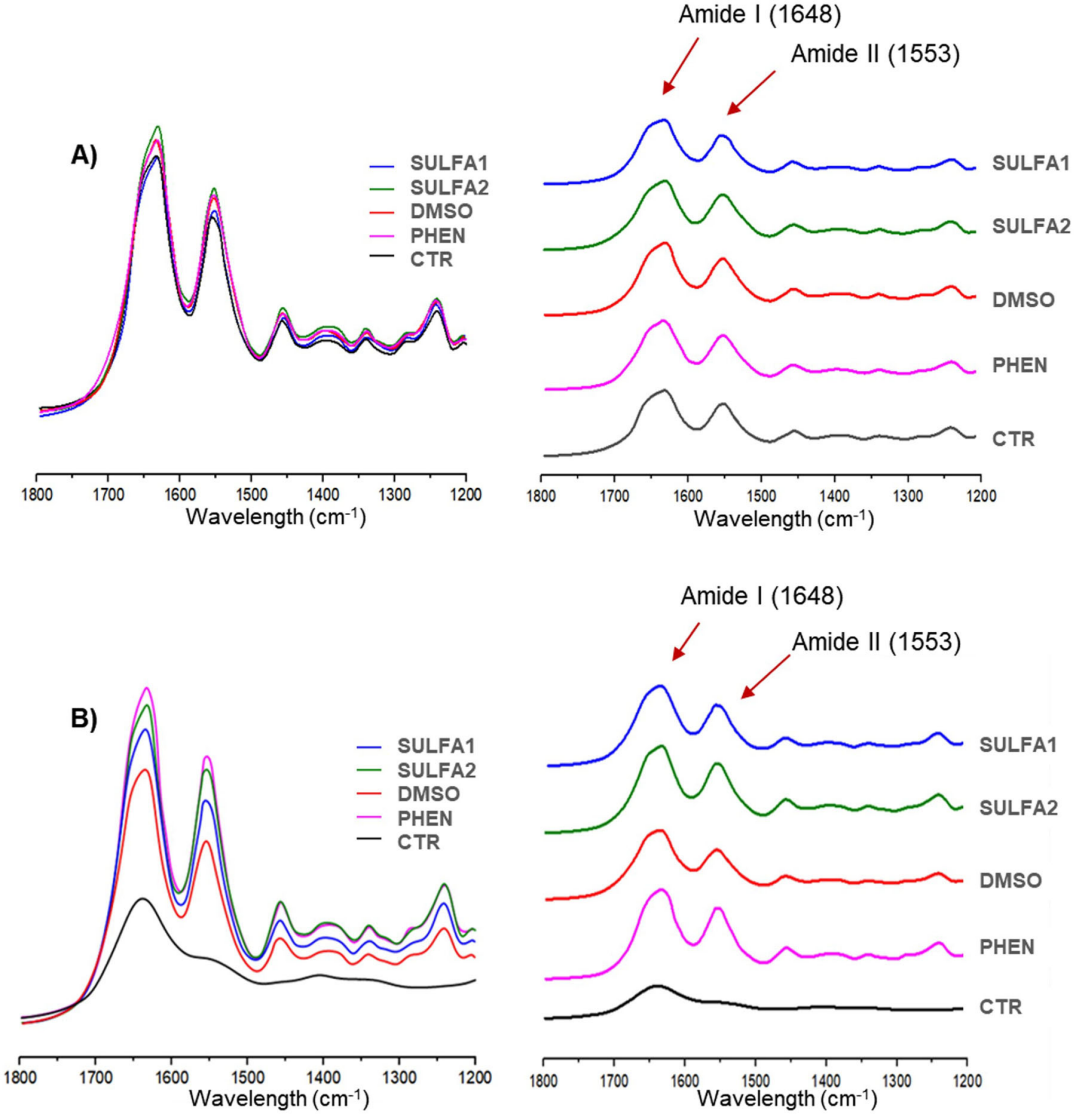
Representative FT-IR spectra after inhibitor application (A) and after storage in collagenase solution for 15 days (B).

Representative FT-IR spectra of collagen fibres from all experimental groups are shown in Figure 4. After treatment of the dentine with the substances tested, all spectra, including the control group (without pre-treatment), showed characteristic bands of amide I (1648 cm^−1^) and amide II (1563 cm^−1^) (Figure 4(A)). After 15 days of storage in collagenase solution (B), the amide II band was observed in all groups except for the CTR group.

### Antibacterial activity of compounds

**Figure 5.**
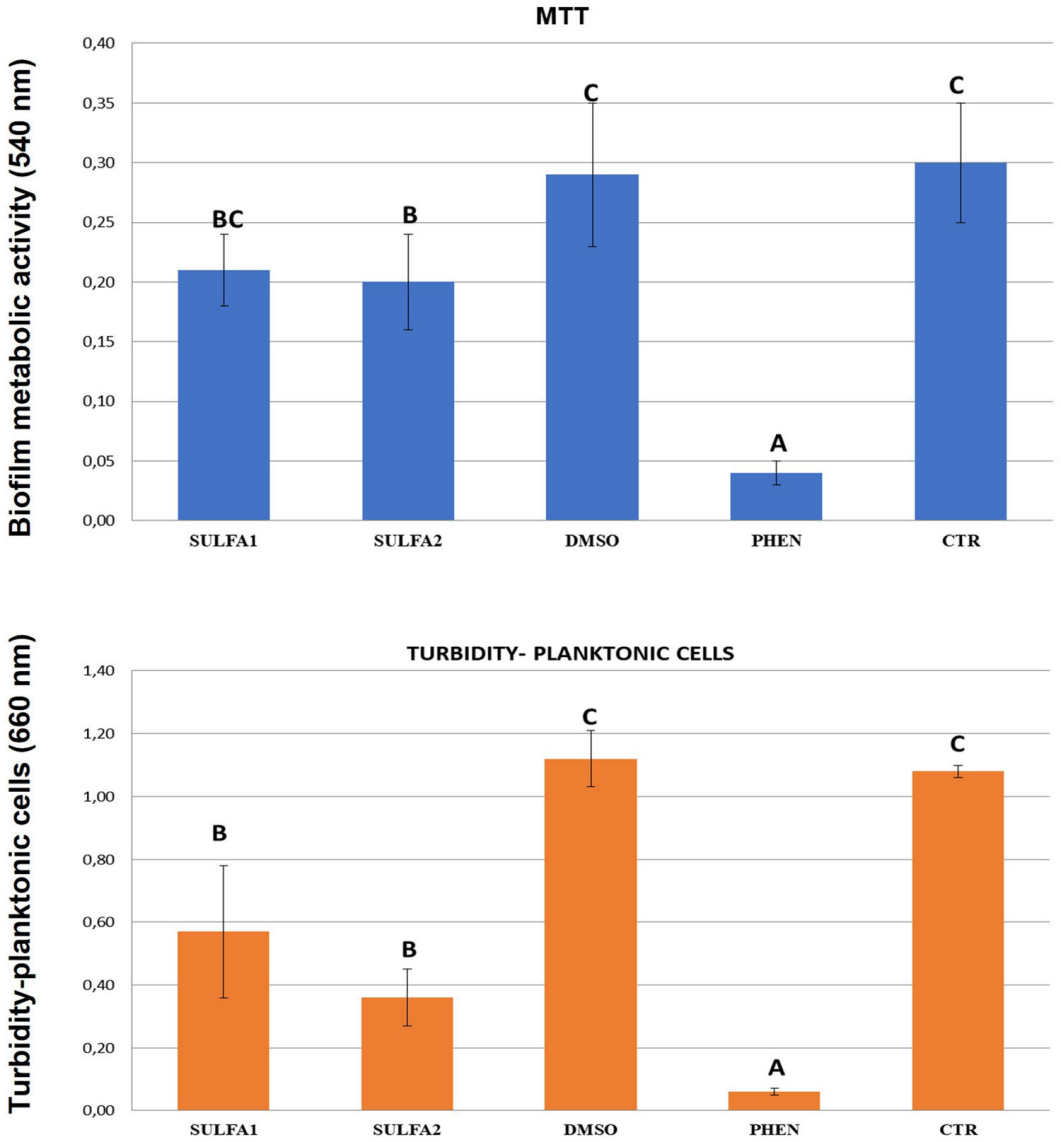
Average values (±DP) for *S. mutans* biofilms in different types of surface treatment: MTT (metabolic activity) and Turbidity (planktonic cells). Slashes by different capital letters are statistically different (HSD of Tukey, *p* < 0.05).

Quantitative results for preformed *S. mutans* biofilms (metabolic activity of biofilm-forming cells) and cell growth (measurement of planktonic cells in the biofilm), in the presence of treated/untreated dentine blocks with SULFA1 and SULFA2, are shown in Figure 5. The treatment influenced biofilm production on dentine surfaces and cell growth (*p* < 0.05). The SULFA2 group had lower values of metabolic activity (lower biofilm production) when compared to the CTR (*p* < 0.05). Both SULFA1 and SULFA2 inhibited the growth of planktonic cells in the suspension used for biofilm induction. The SmuCA inhibitory effects of the two sulphonamides are shown in Figure 1.

## Discussion

The present study aimed to evaluate the effect of dentine pre-treatment with two synthesised sulphonamides CAI derivatives (arylsulphonyl-ureidosulphonamides)^29^^,^^30^ (SULFA1 and SULFA2) on dentine bond strength, dentine surface wettability, collagen preservation of the dentine matrix, and biofilm formation of *S. mutans.* Sulphonamides constitute an important class of drugs with anti-glaucoma, anti-epileptic, diuretic, and antitumor action^29^^,^^30^^,^^39^. Sulphonamides have also been explored as an alternative treatment against oral diseases. These compounds have shown inhibitory activity on CAs and metalloproteases, both present in *S. mutans* and *Porphyromonas gingivalis*, bacteria that cause caries and periodontal diseases, respectively. CAs are essential for developing and replicating these microorganisms, and their inhibition significantly affects bacterial growth^24^^,^^25^. However, to our knowledge, there is no report regarding the inhibitory effect of sulphonamides on the dentinal matrix metalloproteinases. A study of 2000^31^ explored whether primary sulphonamides acting as effective CA inhibitors also inhibit MMPs. Those simple sulphonamides in fact do not inhibit MMPs, but their N-hydroxy analogs do inhibit both classes of zinc enzymes, the CAs and the MMPs. As arylsulphonyl-ureido benzenesulphonamides (as SULPHA 1 and 2)^30^ are an underexplored class of highly effective CA inhibitors, we decided to include them in this study, also considering their interesting inhibitory effects on *S. mutans* CA (see later in the text).

For this study, 2 control groups were used: the positive control group (PHEN) and the negative control group (CTR). Also, it employed an adhesive system already well documented in the literature: the Adper Single Bond 2^40^^,^^41^. The group with the phenanthroline inhibitor was used as a positive control since there is scientific evidence that proves its Effectiveness in inhibiting MMPs^42–45^, also corroborating the results obtained in this study in the evaluation of degradation of collagen (Figure 4(B)). 1,10-Phenanthroline is one of the most successful and extensively studied heterocyclic ligands, exhibiting activity against many microorganisms due to its ability to chelate essential metals, interfering with their acquisition and bioavailability for crucial metabolic reactions^42^. This inhibitor forms very stable complexes with Zn^2+^ and, to a lesser extent, with Fe^2+^, Ca^2+^, Cu^2+^, Co^2+^, and Mn^2+^^43^. As expected, the chelation of essential metallic elements impairs the functioning of various biological systems and disrupts microbial cell homeostasis, culminating in various metabolic dysfunctions, including cell death^44^^,^^45^. Therefore, this chelator cannot be used as a human metalloproteinase inhibitor due to its high cytotoxicity.

According to the dentine bond strength results obtained in this study, SULFA1 maintained its activity after 12 months of storage in water and after the cariogenic challenge, while SULFA2 showed an increase in the bond strength value. Thus, hypothesis 1 may be considered as partially validated since pre-treatment of the dentine surface with sulphonamide derivatives maintained or increased micro tensile bond strength after 12 months and after the cariogenic challenge.

The sulphonamide derivatives tested in the present study (SULFA1 and SULFA2) were solubilised in dimethyl sulphoxide. Therefore, a control group with this solvent was used since Tjäderhane et al^46^. and Cardenas et al^47^. observed that using an aqueous solution with DMSO as a dentine pre-treatment was able to preserve bond strength over 12 months. Probably, this effect was observed due to the ability of DMSO to dissociate highly cross-linked collagen into a more dispersed network of apparent fibrils, breaking the auto-associative tendency of water and consequently improving wettability in demineralised dentine^46^. In the present study, this same effect was observed since the 12-month bond strength value of the DMSO group was maintained compared to the initial value. However, when compared with the experimental groups SULFA1 and SULFA2, the results of bond strength were statistically lower at 24 h, after 12 months, and after exposure to a biofilm of *S. mutans* (cariogenic challenge), suggesting that the use of DMSO did not enhance the bond strength results of SULFA1 and SULFA2.

Dental caries is a dynamic multifactorial disease that requires a microbial biofilm to act on a suitable substrate. One of the microorganisms widely involved in developing this pathology is *S. mutans*, a gram-positive bacterium in the oral cavity. When installed on the dental surface and associated with other factors, such as diet and host susceptibility, these bacteria produce acids that promote a pH drop to below 5.5, diffusing through mineralised dental tissues. Consequently, the mineral portion dissolves and exposes the collagen fibres. These unprotected fibres are degraded by the action of MMPs that are activated by low pH^5^^,^^12^^,^^48^. Due to this, one of the ways to evaluate the effect of pre-treatment of these substances (SULFA1 and SULFA2) on dentine adhesion strength was the ageing of beams in a biofilm of *S. mutans*. It was observed an increase in bond strength in the SULFA2 group and maintenance of microtensile bond strength in SULFA1 compared to the other groups after the cariogenic challenge. This can be explained by the inhibitory action of the sulphonamide derivatives tested on MMPs and the antimicrobial effect of SULFA1 and SULFA2 observed in the present study.

The failure pattern described in Figure 2 shows that the SULFA1 group presented a predominance of mixed failures followed by a significant increase in cohesive failure in resin after 12 months in distilled water. This result suggests that the tested substances could maintain the integrity of the dentine/adhesive interface, thus failing in the resin body. SULFA2, DMSO, PHEN, and CTR groups, in 24 h, 12 months, and after cariogenic challenge, presented mainly mixed failure. However, for the PHEN group, it was also possible to observe the predominance of adhesive failure at 24 h and after cariogenic challenge.

Since adhesion requires intimate contact between the adhesive material and the substrate, the dentine surface’s wetting process directly influences the adhesive interface’s quality. The degree of propagation of a liquid over a surface is the measure of wettability, which can be quantified by determining the contact angle^15^. For the use of conventional adhesive systems, whether simplified or not, acid etching dissolves the smear layer, which is removed by subsequent washing, allowing direct contact of the adhesive with demineralised dentine and, as a result, better surface wettability^15^. Given the above, a treatment after acid etching would negatively affect the contact angle. Therefore, considering the different dentine treatments evaluated, the SULFA1 and SULFA2 groups led to contact angles statistically equal to the CTR group, suggesting that the treatment with these inhibitors could not change the penetration of the adhesive and the formation of the hybrid layer. Thus, the first hypothesis was entirely accepted since sulphonamide derivatives did not influence dentine wettability. These results may be associated with the presence of -NH endings in the SULFA1 and SULFA2 groups, which allow the interaction with the water molecules present in the demineralised dentine through hydrogen bonds^49^, in addition to interacting with the hydrophilic endings of the adhesive monomers, allowing a better wettability on the substrate pre-treated with the derivatives.

For the spectroscopic analysis of collagen degradation, treatment groups were compared to the PHEN group. After qualitative analysis of the amide I and amide II curves, it was observed that, like phenanthroline, the aryl sulphonyl-ureido sulphonamide derivatives (SULFA1 and SULFA2) at a concentration of 200 µM were also able to reduce collagen degradation (Figure 4(B)) after 15 days in collagenase solution. It may be suggested that the sulphonamide derivatives tested could interact with the active site of MMPs, allowing the inactivation of these enzymes. Therefore, the second hypothesis was accepted concerning the collagen preservation capacity of the dentine matrix by inhibiting the proteolytic activity of collagenases by aryl sulphonyl-ureido sulphonamide derivatives.

According to Dedeoglu et al^50^^,^^51^, sulphonamides have antimicrobial action against *S. mutans* due to the inhibition of bacterial CAs present in this pathogen. Considering these data, the ability of the sulphonamide derivatives to inhibit cell growth and biofilm formation of *S. mutans* on dentine blocks was tested. It was observed that both compounds decreased the number of planktonic cells, and SULFA2 decreased the rate of metabolic activity of the cells in the biofilm formed in the dentine blocks. When trying to extrapolate these results to the daily clinic, we can assume that using these sulphonamide derivatives may minimise or hinder the development of recurrent caries at the margins of adhesive restorations. Additionally, we can accept the third hypothesis since the sulphonamide derivatives showed antimicrobial and antibiofilm activity of *S. mutans*. Indeed, both sulphonamides are potent SmuCA inhibitors, with inhibition constants in the range of 173–215 nM (Figure 1), which means that they probably interfere with the pathogen’s growth, as already shown for other bacteria and sulphonamide inhibitors^52–68^.

In vitro studies have already evaluated the cytotoxicity of these sulphonamide derivatives against different animal cells, showing slight toxicity against the macrophage cells RAW 267.4^69^ and lower toxicity than amphotericin B against human red blood cells^70^. Although we could extrapolate these results to the oral cavity, more specific cellular models should be used in future research.

## Conclusions

Based on current data, we can conclude that pre-treatment with the arylsulphonyl-ureido sulphonamide derivatives SULPHA 1 and SULFA2 preserved the adhesive interface even after a cariogenic challenge with *S. mutans*. It did not interfere negatively with the wettability of the dentine surface, and this result is vital since wettability directly influences the quality of the adhesive interface. They could inhibit MMPs preserving collagen and could still reduce the metabolic activity of *S. mutans*. Given these results, SULFA1 and SULFA2 could offer improvements in the performance of adhesive systems, allowing a reduction in recurrent risks or the development of recurrent caries at the margins of composite restorations due to their inhibitory action against the bacterial CA present in *S. mutans*. However, a broader approach to the subject is still needed regarding the use of SULFA1 and SULFA2 in an adhesive protocol or the incorporation of these compounds into experimental adhesive systems, as well as a comparative study with MMP inhibitors already known (e.g. chlorhexidine).
